# A Study to Evaluate the Lifestyle of Medical Students in Lahore, Pakistan

**DOI:** 10.7759/cureus.4328

**Published:** 2019-03-27

**Authors:** Usama Nasir, Ayesha Farooq Butt, Sarah Choudry

**Affiliations:** 1 Internal Medicine, Combined Military Hospital Lahore Medical College and Institute of Dentistry, Lahore, PAK

**Keywords:** healthy lifestyle, students, medical students, college, adolescents

## Abstract

Introduction

Medical school is a challenging period that may bring about undesired changes in health and lifestyle habits. In order to thrive both mentally and physically, students must maintain a balanced routine and adopt healthy lifestyles. Scientific evidence confirms that unhealthy life habits play an important role in the development of many disorders in all age groups. Our aim was to evaluate the prevalence of these lifestyle habits among medical students of a renowned private sector medical school based in Lahore, Pakistan, and to identify their association with variables such as socio-demographic characteristics, year of medical school, and personal beliefs.

Methodology

This cross-sectional study of 300 medical students included a self-administered questionnaire comprising (in addition to socio-demographic characteristics) information about healthy lifestyle practices in the following areas: gender, age, sleep, dietary habits, addictions, physical activity, and hygiene practices. The students were undergraduates in the first to fifth years of M.B.B.S, aged ≥18 years, and gave informed consent. Data were analyzed using SPSS 16 (IBM Corp, Armonk, NY, US). The chi-squared test was used to determine the association.

Results

The frequencies of healthy, fairly healthy, and unhealthy lifestyles were 30.7%, 62.3%, and 7%, respectively. In a sample of 33.3% males and 66.7% females, the fairly healthy lifestyle was most prevalent (62.3%) followed by the healthy lifestyle (30.7%) and unhealthy lifestyle (7.0%). Third-year MBBS had the highest prevalence of healthy lifestyle (n=20, 40.8%). Fourth-year MBBS had the highest prevalence of a fairly healthy lifestyle (n=70, 75.3%). Whereas, second-year MBBS had the highest prevalence of unhealthy lifestyle (n=9, 11%). This difference between classes was statistically significant (p=0.006).

Conclusion

In order to combat the low level of healthy lifestyles in medical students, it is recommended that measures such as awareness programs and seminars be put in place.

## Introduction

University is a period of responsibility in terms of choices and lifestyle practices [1], where students are exposed to the challenges of young adulthood while simultaneously tackling the mental and social issues of student life [2]. University students represent the future of families, communities, and countries. As a consequence, they also face the stresses of achieving success in their academic goals [3] while being expected to be competitive, adding to demands and burdens, which can lead to further stress [4]. Many students confront changes in living conditions and health-promoting/damaging adjustments to lifestyle and environment [5]. In a recent study, it was noted that the average weight gain of freshmen during the first term of university was 1.3-3.1 kg [6]. It is, therefore, postulated that university students, considering the rigorous curriculum they are put through, must adapt to healthy lifestyles in order to thrive both physically and mentally.

It is well-known that a healthy lifestyle is of benefit not only in the prevention of disease but also in the promotion of well-being [7]. Unhealthy lifestyle behaviors, particularly poor dietary practices, physical inactivity, and smoking can often lead to problems such as obesity and chronic non-communicable diseases. Poor dietary habits and smoking are major causes of both cardiovascular disease and cancer. Healthy practices, on the other hand, such as weight management, physical recreational activity, and proper sleeping habits can have a positive impact on health status. Numerous studies have indicated sex differences in health behavior. Among university students, women were found to demonstrate more positive health behavior, higher awareness, and stronger beliefs concerning the importance of positive health habits [8].

The prevalence of healthy activities amongst medical students is of even more importance, as they will become future physicians, and students who deliberately overlook the importance of a healthy lifestyle are more likely to fail in establishing health promotion opportunities in their patients. Furthermore, medical students have been shown to exhibit early risk factors for chronic diseases [9], thus reiterating the fact that maintaining healthy lifestyles is of the utmost importance in this demographic.

Over the past decade, it has become apparent that chronic physical activity in the form of exercise training has the ability to prevent or delay the onset of illness and disease [10]. Lifestyle choices with respect to diet are important in both the primary and secondary prevention of chronic disease [11].

The objective of this study, while keeping the aforementioned studies in mind, was to determine the prevalence of healthy activities in medical students. This study broadly aimed at scrutinizing the commonly present risk factors and unhealthy acts among students. Since no significant studies have been documented regarding this topic in Pakistan, this research will serve as statistical evidence to signify the importance of this matter in an attempt to improve the overall health status of students throughout the country.

## Materials and methods

Study approval was obtained from the Ethics Review Committee, Combined Military Hospital (CMH), Lahore Medical College. A cross-sectional study design was used. The data collection form had 15 questions in total, as shown in the figure in the Appendix. The first three questions addressed basic demographics. The remaining 12 questions were about lifestyle practices and included questions about smoking status, consumption of fruits and vegetables, adequate hours of sleep per day, amount of sugar and salt in the diet, alcohol consumption, dental hygiene, hand hygiene before food consumption, aerobic exercise, and daily activity level. The results were categorized into three sets based on the responses to the questionnaire. Students following one to four healthy lifestyle practices were placed in the unhealthy lifestyle set. Students with five to eight healthy practices were paced in the fairly healthy lifestyle set, and those with nine or more were placed in the healthy lifestyle set.

This questionnaire was then pilot-tested on 25 students of CMH Lahore Medical College, yielding a mean time required to fill the questionnaire of approximately four minutes. It was then distributed randomly to a total of 350 MBBS students throughout the medical school. Of these, 314 forms were returned (response rate = 89.71%). Data were collected by the authors and informed consent was verbally obtained. Fourteen of the 314 forms were discarded due to faulty or incomplete entries, and data from 300 forms was entered.

Data were analyzed in SPSS v. 16 (IBM Corp., Armonk, NY, US). The chi-square test was used to analyze correlations between categorical variables. Descriptive statistics, such as frequencies and percentages, were calculated for qualitative variables. The chi-square test of significance was used to determine the association between gender and lifestyle and medical school year and lifestyle. Continuous variables were represented by the respective value and standard error of mean (SEM). A p-value <0.05 was considered statistically significant.

## Results

**Figure 1:**
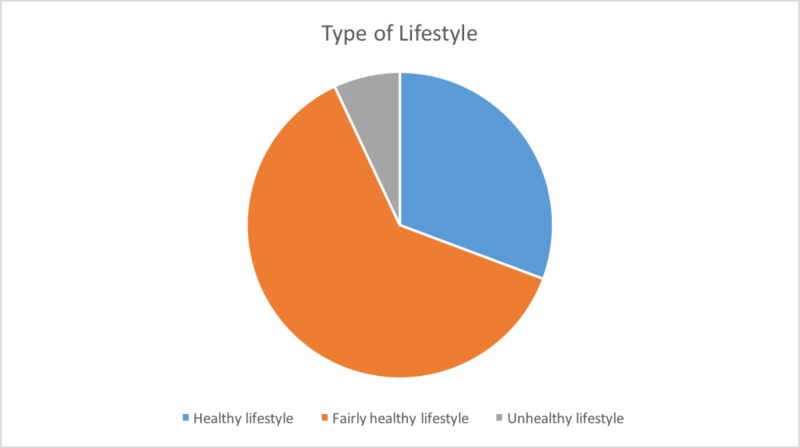
Prevalence of different lifestyles

There were 300 respondents in total. Of these, 100 were males (33.3%) and 200 were females (66.7%). Among all the students, the fairly healthy lifestyle was most prevalent (n=187, 62.3%), followed by the healthy lifestyle (n=92, 30.7%) and unhealthy lifestyle (n=21, 7%). This is shown in Figure 1.

The highest number of students in the healthy lifestyle category belonged to second-year MBBS (n=32, 34.8%). Among the students with a fairly healthy lifestyle, the majority (n=70, 37.4%) belonged to fourth-year MBBS. Whereas, in the unhealthy lifestyle category, the most number of students were from second-year MBBS (n=9, 42.9%). This is shown in Table 1.

**Table 1:** Prevalence of lifestyles according to medical school year

	Healthy lifestyle n (%)	Fairly healthy lifestyle n (%)	Unhealthy lifestyle n (%)	Total
First-year MBBS	10 (10.9%)	30 (16.0%)	1 (4.8%)	41
Second-year MBBS	32 (34.8%)	41 (21.9%)	9 (42.9%)	82
Third-year MBBS	20 (21.7%)	28 (15.0%)	1 (4.8%)	49
Fourth-year MBBS	16 (17.4%)	70 (37.4%)	7 (33.3%)	93
Fifth-year MBBS	14 (15.2%)	18 (9.6%)	3 (14.3%)	35
Total	92	187	21	300

**Figure 2:**
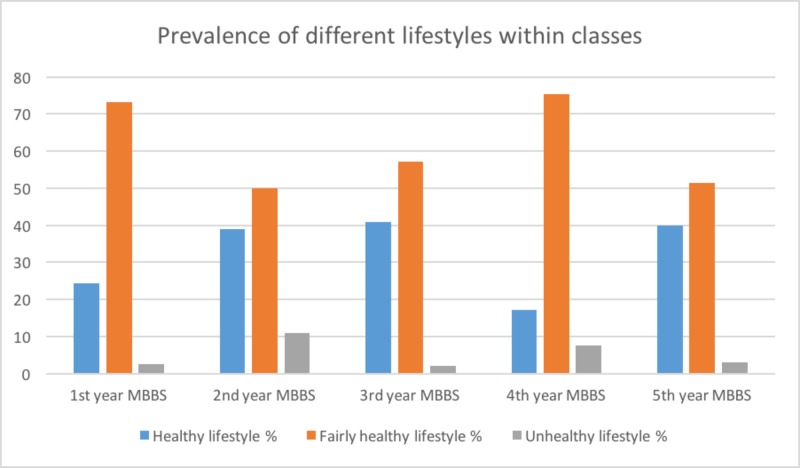
Prevalences of different types of lifestyles within classes

Third-year MBBS had the highest prevalence of the healthy lifestyle (n=20, 40.8%). Fourth-year MBBS had the highest prevalence of the fairly healthy lifestyle (n=70, 75.3%). Whereas, second-year MBBS had the highest prevalence of unhealthy lifestyle (n=9, 11%). This difference in lifestyle between classes was found to be statistically significant (p-value=0.006). These results are also listed in Figure 2.

With regards to gender distribution of lifestyle, the healthy lifestyle was more prevalent (n= 67, 33.5%) in female students as compared to male students (n=25, 25%). However, this difference was not statistically significant (p=0.079). This is shown in Table 2.

**Table 2:** Prevalence of various grades of lifestyle according to gender

Lifestyle	Gender		Total
	Male n (%)	Female n (%)	
Unhealthy	11 (11%)	10 (5%)	21
Fairly healthy	64 (64%)	123 (61.5%)	187
Healthy	25 (25%)	67 (33.5%)	92
Total	100	200	300

## Discussion

The purpose of this study was to understand the lifestyle practices of Pakistani medical students. The major limitation of this research was the fact that all aspects of a healthy lifestyle were not covered. Future research could be carried out focusing on the remaining details including hours of study, a mental health/stress quotient, etc. The sample size and demographics of this research also posed as limitations. Further studies should be carried out with more participants, not only in number but also distributed among different medical schools. Along with widening the scope of this research, it would also allow for a more comprehensive assessment of the topic.

College life is an important stage for individuals because the element of "behavior" is conducive to change during this time. University and college arenas, therefore, represent an important opportunity for health and nutritional education. Unhealthy habits picked up at this level generally persist throughout adult life [12]. As mentioned previously, college life is also a period during which individuals are, for the most part, exposed to stress and lack of time, posing a barrier to the adoption of healthy practices. In a documented survey about the habits and perceived barriers to following a healthy lifestyle in a college population, the biggest deterrent to exercise and good eating habits was “lack of time” (36%) [13].

In recent studies, the above-mentioned factors have been well documented. Citing a study in Japan, almost half of the dental students missed one of the three main meals [14]. In another cross-sectional survey in the UAE, a large percentage of medical students were found to be either underweight or obese and most were found to believe that their activity levels were insufficient, stress levels too high, and diets unhealthy [15]. A pilot study on medical students in Poland showed non-adherence to advise regarding a healthy lifestyle with regard to sleep, food and fluid intake, and exercise [16]. A local study on the medical students of the Aga Khan University, Pakistan, showed that the majority (>90%) of students thought they had been stressed at one time or another, which affected not only their academic performances but also all aspects of health [17].

In the research (which included the data collected by the World Health Organization (World Health Survey, 2003) and Inner-City Fund (ICF) Macro International and the Ghana Statistical Service (Ghana Demographic and Health Survey, 2008)) carried out at the University of Ghana, the results of 4916 females and 4568 males were evaluated. The prevalence of some negative lifestyle behaviors, such as smoking, had reduced while others such as alcohol consumption had increased. Relatively fewer people adhered to consuming the recommended amount of fruit and vegetable servings per day in 2008 as compared to 2003. While more females (7.0%) exhibited healthier lifestyles, more males (9.0%) exhibited risky lifestyle behaviors [18]. Similarly, our research also found that more female students (22.3%) exhibited healthy lifestyles as compared to their male counterparts (8.3%).

A recent longitudinal study done by Stephens et al. used objective measures of physical fitness to assess medical students at the Uniformed Services University [19]. Their results indicated that individual levels of physical fitness declined during medical school, most notably during the preclinical years. The result of our study shows no such pattern relating to the lifestyle of medical students throughout the five years of medical school.

It is unsurprising that the results of several studies suggest that the introduction of nutritional and physical activity topics in medical students’ curriculum is needed to positively influence both knowledge and behaviors [20-21]. However, it should also be noted that in our research, while we found that the majority of students were aware of basic factors affecting lifestyle, they still found it difficult to implement the necessary changes. Therefore, it is necessary that further research be carried out in this area in order to not only identify but also address these perceived barriers.

It is a well-established fact that healthy lifestyles not only prevent disease but also promote a sense of well-being. In order to attain this, certain goals must be achieved. It is fitting to highlight here the healthy lifestyle recommendations given out by the WHO. These guidelines emphasize the role of fruits and vegetables in the diet and recommend eating at least 400 g, or five portions, of fruit and vegetables per day. They also recommend the reduction of fat to no greater than 30% of the daily caloric value - primarily coming from unsaturated vegetable oils or soft margarine instead of saturated fats. In addition to this, milk and dairy products that are low in both fat and salt should be used while refined sugar, sugary drinks, and sweets should only be used sparingly. Total salt intake should not be more than one teaspoon (5 g) per day, including the salt in bread and processed cured and preserved foods. Alcohol and smoking in any quantity should be avoided, and a conscious attempt should be made to keep the body well-hydrated. The homeostatic balance of the body also includes a regularity of the circadian rhythm and a balance of stress and anti-stress hormones. It is important, therefore, to get a minimum of seven hours of sleep per night and maintain positive mental well-being by avoiding stress [22].

## Conclusions

Keeping in view the medical background of the sampled population, the prevalence of a healthy lifestyle is quite low (31%). Efforts are needed in order to help students implement these healthy lifestyle practices. It is suggested that measures such as awareness programs, seminars, and health fairs be explored.

## Figures and Tables

**Figure 3:**
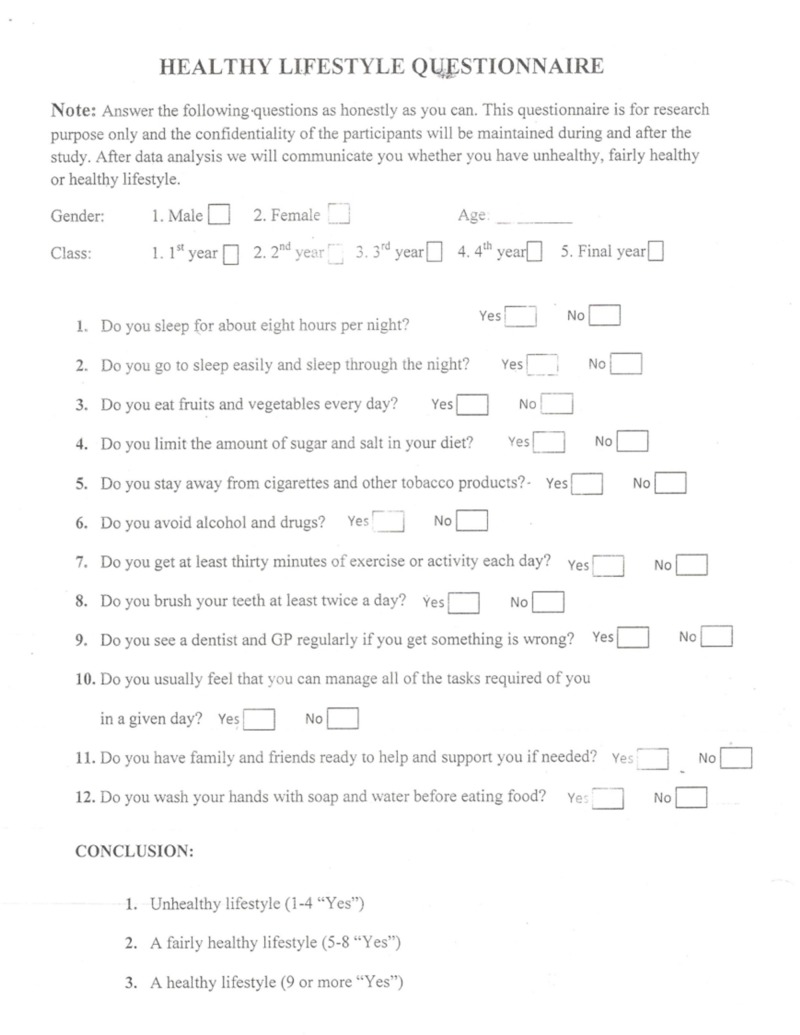
Questionnaire
